# Delineation of condition specific *Cis*- and *Trans*-acting elements in plant promoters under various Endo- and exogenous stimuli

**DOI:** 10.1186/s12864-018-4469-4

**Published:** 2018-05-09

**Authors:** Chi-Nga Chow, Yi-Fan Chiang-Hsieh, Chia-Hung Chien, Han-Qin Zheng, Tzong-Yi Lee, Nai-Yun Wu, Kuan-Chieh Tseng, Ping-Fu Hou, Wen-Chi Chang

**Affiliations:** 1 College of Biosciences and Biotechnology, Institute of Tropical Plant Sciences, National Cheng Kung University, Tainan, 701 Taiwan; 2 School of Science and Engineering, The Chinese University of Hong Kong, Shenzhen, China

**Keywords:** Abiotic stress, *Cis*-acting elements, Co-expression, Hormone, Microarray, Transcription factors

## Abstract

**Background:**

Transcription factors (TFs) play essential roles during plant development and response to environmental stresses. However, the relationships among transcription factors, *cis*-acting elements and target gene expression under endo- and exogenous stimuli have not been systematically characterized.

**Results:**

Here, we developed a series of bioinformatics analysis methods to infer transcriptional regulation by using numerous gene expression data from abiotic stresses and hormones treatments. After filtering the expression profiles of TF-encoding genes, 291 condition specific transcription factors (CsTFs) were obtained. Differentially expressed genes were then classified into various co-expressed gene groups based on each CsTFs. In the case studies of heat stress and ABA treatment, several known and novel *cis*-acting elements were identified following our bioinformatics approach. Significantly, a palindromic sequence of heat-responsive elements is recognized, and also obtained from a 3D protein structure of heat-shock protein-DNA complex. Notably, overrepresented 3- and 4-mer motifs in an enriched 8-mer motif could be a core *cis*-element for a CsTF. In addition, the results suggest DNA binding preferences of the same CsTFs are different according to various conditions.

**Conclusions:**

The overall results illustrate this study may be useful in identifying condition specific *cis*- and *trans*- regulatory elements and facilitate our understanding of the relationships among TFs, *cis-*acting elements and target gene expression.

**Electronic supplementary material:**

The online version of this article (10.1186/s12864-018-4469-4) contains supplementary material, which is available to authorized users.

## Background

A series of gene regulation is critical for plant to adapt the environmental changes [1–3]. The regulation of spatial and temporal transcription via transcription factors (TFs) derives plant survival with short- and long- term impacts on plant physiology and development [4, 5]. Thus, identification of TF binding sites on promoter sequences of their target genes is essential to characterize TF function [6].

In the past few years, several computational and experimental methods have been used to identify the relationship between TFs and *cis-*acting elements. Some useful resources have been also developed to construct transcriptional regulatory networks, such as JASPAR and AGRIS [7, 8]. However, the DNA binding information of most TFs is still very limited. For example, among 1717 *Arabidopsis* TFs from PlantTFDB, only 64 TFs and three TF complexes with their target genes have been characterized. Furthermore, the corresponding TFs of several *cis*-acting elements collected in recent databases are not available [7–12]. Recently, protein-binding microarrays are applied to study the DNA binding sequences of 63 and 313 TFs from *Arabidopsis* in two previous studies, respectively [13, 14]. Other methods such as chromatin immunoprecipitation combining with sequencing (ChIP-seq) and microarray (ChIP-chip) are also broadly employed in TF binding sites discovery under a given condition. For example, ABA-elicited transcriptional regulation for 21 ABA-related TFs were constructed by using ChIP-seq and RNA sequencing [15]. Moreover, DNA affinity purification sequencing (DAP-seq) was introduced to investigate the *cis-*acting elements and DNA modifications pattern for 529 (30%) *Arabidopsis* TFs [16].

In addition, some studies indicate that co-expressed members shared similar biological functions, which suggest a potential framework of transcriptional regulation [17, 18]. This concept has been widely used to investigate the functional elements involved in regulating transcriptional activity [19–21]. For instance, several functional *cis*-acting elements have been identified as key regulatory components related to the stress or pathogen-responsive pathways on the basis of gene expression clusters [22].

Although these researches illustrate the possibility of genome–wide analysis, current understanding of the relationships among TFs, *cis-*acting elements and target gene expression is still limited. In this work, we have developed a new bioinformatics approach for identification of condition specific *cis*- and *trans*- regulatory elements by using microarray expression data and genomic promoter sequences. Based on 344 *Arabidopsis* microarray samples, 291 condition specific TFs (CsTFs) were defined. Furthermore, the potential *cis*-acting elements of each CsTF were examined to reveal possible regulatory map of the CsTF. The results of the enriched 8-mer motif analysis from co-expressed clusters exhibit high consistency with the known condition responsive *cis*-acting elements.

## Methods

### Microarray data collection and processing

The *Arabidopsis thaliana* microarray data were obtained from the AtGenExpress and the NASCArrays [23, 24]. A total of 344 microarray samples including nine abiotic stresses (cold, osmotic, salt, drought, genotoxic, UV-B, wounding, heat, and oxidative) extracted from shoot and root and eight hormones treatments (IAA, cytokinin, gibberellin, brassinolide, ABA, methyl jasmonate, GA-3 and ACC) were used. The platform for all samples was Affymetrix ATH1 microarray chip (GPL198). For all conditions, two replicate samples were analysed. Array intensities were background adjusted and quantile normalized by using the justRMA function in the *affy* package of Bioconductor in R statistical language [25, 26]. The probe set annotation table downloaded from TAIR database (v10) was used to identify corresponding genes of each probe set ids [27]. Any ambiguous probe set ids which were associated with more than two genomic loci were discarded. A total of 20,922 genes were applied for further analysis.

### Identification of condition specific transcription factors

A list of *Arabidopsis* TF-coding genes and their regulatory information (i.e. experimental binding matrices and annotation) were retrieved from PlantTFDB and PlantPAN 2.0 [9, 28]. Among 1717 TFs from PlantTFDB, only 1367 TFs could be identified in GPL198 platfrom. To recognize CsTFs for each condition, differentially expressed TFs (DETFs) were selected by using Students *t*-test between control and treatment with the confidence interval 0.99 and log_2_ fold change large than 1. The *p*-value of Students *t*-test statistic method was performed by using t.test () function in R. Furthermore, the z-score of fold change were used as a measurement to choose CsTFs from DETFs. The formula is as follows:

$$ {z}_{condition\ a}=\frac{x-\mu }{\sigma } $$

where *condition a* denotes the corresponding condition what a TF is defined as DETF, whereas *x* is the fold change values in the treatment relative to control for *condition a*. μ and σ are mean and standard deviation of the fold changes for all conditions in the dataset of *condition a*, respectively. Please see Additional file 1: Table S1 for the treatment, control, and dataset of each condition. Totally, 291 DETFs were deified as CsTFs with z-score larger than 2.

### Co-expressed gene groups of CsTFs

To identify the co-expression gene groups, differentially expressed genes (DEGs) (*P* < 0.01; |log_2_ (fold change)| ≥ |log_2_ (1.5)|) were selected from the same induced condition of a CsTF. Then, the expressions data of all samples from the corresponding dataset were used to assess co-expression between a CsTF and a DEG (in Additional file 1: Table S1). The co-expressed genes of each CsTF were selected from the DEGs based on Pearson correlation coefficients (PCC ≥ 0.8). The PCC was calculated by using the cor () function in R.

### Construction of genomic promoter dataset for *Arabidopsis* genes

A promoter was defined as the 1000 bp upstream of the transcription start site. All promoter sequences as well as the transcription start sites of 41,671 *Arabidopsis* transcripts (33,602 genes) were obtained from the TAIR [27]. To refine promoter sequences, we eliminated 25 promoters that contain uncertain bases (i.e. N, S, K, M, R, and W). Among 33,577 genes with annotated promoters, 5884 (18%) genes have multiple transcripts with the same TSSs or the TSSs closed to each other. Since the probe annotation of GPL198 is based on detectable genes rather than transcripts, promoters from different transcripts should be merged to avoid multiple calculation of one motif. We merged their promoter sequences according to their locations in the genome (Additional file 1: Figure S1). Finally, 33,715 promoter sequences for 33,577 genes were constructed as genomic promoter dataset for *Arabidopsis*.

### Enrichment analysis of *cis*-acting elements in condition-specific promoters

The *cis*-acting elements of a CsTF were identified from promoter regions of its co-expressed gene group. To construct background calibration required for motif enrichment analysis, all possible 4–8-mer motifs (87,296 motifs) were scanned in the genomic promoter dataset of *Arabidopsis*. Since many motifs can be found in one promoter many times, both the presence/absence and frequency of motifs were taken into consideration. We thus evaluate motif enrichment using the following two criteria:

“Presence enrichment” assesses whether a motif significantly arise in a co-expressed gene group by comparing to whole genome background. As such, for each motif (*motif a*) assigned to a given CsTF its probability (*p*-value) was calculated by using a hypergeometric distribution, based on the following formula:

$$ {p}_{motif\ a}=\sum \limits_{i=x}^M\frac{\left(\begin{array}{c}M\\ {}i\end{array}\right)\left(\begin{array}{c}N-M\\ {}n-i\end{array}\right)}{\left(\begin{array}{c}N\\ {}n\end{array}\right)} $$

where *x* is the number of co-expressed genes whose promoters contain *motif a*, *n* is the number of co-expressed genes, *N* is the total number of genes in the background population, and *M* is the number of genes whose promoters contain *motif a* in the background set.

“Number enrichment” is used to estimate the preferred frequency of one motif in the promoters of the co-expressed gene group related to a CsTF in comparison with the background promoter sets. Different to presence enrichment, number enrichment for *motif a* was computed using the above formula of hypergeometric distribution where *x* is the number of *motif a* resides in the promoters of co-expressed genes, *n* is the number of respective bases for *motif a* in the promoters of co-expressed genes, *N* is the total number of respective bases for *motif a* in the background population, and *M* is the number of *motif a* in the background promoter sequences. An example of calculating “presence enrichment” and “number enrichment” is illustrated in Additional file 1: Figure S2.

Identification of all possible 4–8-mer DNA motifs in the promoter regions was performed with Bowtie [29]. In both cases, the *P*-value of a motif lower than 0.001 is defined as significantly enriched. The dhyper () and phyper () functions in R were used to obtain the hypergeometric *P*-values.

### Motif alignments and sequence logos of each CsTF

To clarify the DNA binding sequences of a given CsTF, position specific scoring matrices were utilized to describe the frequency of each base at a certain position. In case study of heat stress and ABA treatment, the critical *cis*-acting elements for heat stress and ABA responsiveness have a highly overrepresented 3-mer and 4-mer in the enriched 8-mer motifs, respectively (in Additional file 2: Table S2-S3). Therefore, we designed three steps to discover DNA binding sequences of a given CsTF under all conditions:

- *Step 1: collection of enriched 8-mer motifs with top 10 3-mer and 4-mer motifs*

Eight-mer motifs with both presence enrichment and number enrichment were defined as enriched motifs. Subsequently, the occurrences of 3-mer and 4-mer in all enriched 8-mer motifs were calculated and ranked. The enriched 8-mer motifs containing the 3-mer and 4-mer with top10 frequencies were collected.

- *Step 2: assembly of enriched 8-mer motifs on their original promoters*

All enriched 8-mer motifs collected above were mapped back to the promoter regions of the co-expressed genes. If the enriched 8-mer motifs overlap with others at least one base, they will be merged. Then, we collected the sequences from each overlapping region.

- *Step 3: generation of sequence logos*

The position specific scoring matrices were created based on the alignments of the sequences from the step 2. Multiple sequence alignment of the overlapping motifs was performed to check conserved bases by using the ClustalW program (version 2.1) [30]. The sequence logos for consensus DNA-binding sites were then displayed using the WebLogo tool [31].

Top10 3-mer and 4-mer motifs and sequence logos of each CsTF on all other conditions can be retrieved from our online database (http://wcchang.itps.ncku.edu.tw/CsTFAnalysis/). The motif-motif similarity between CsTFs and Plant Cistrome Database were estimated by using the Tomtom program [16, 32].

### Expression and genomic sequence resources for rice

The microarray expressions data including 276 samples for six hormones treatments were downloaded from RiceXPro [33]. A list of rice TF-coding genes were retrieved from PlantTFDB [9]. Additional file 1: Table S4 illustrates the treatment, control, and dataset of each condition. Promoter sequence for rice were downloaded from RAP-DB [34]. All analysis was used the same analytical flow and cut-off values as above.

## Results

**Fig. 1:**
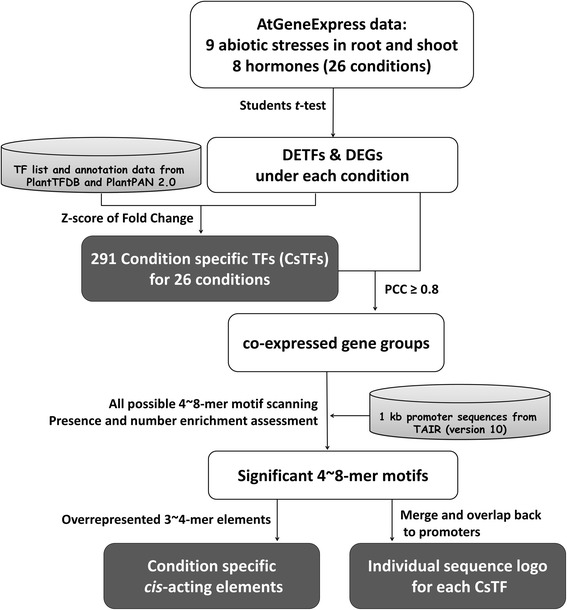
A schematic of the analysis processes to identify significant *trans*- and *cis*- acting elements. TF: transcription factor; DETF: differentially expressed TF; DEG: differentially expressed genes; PCC: Pearson correlation coefficient

The system flow of this research is shown in Fig. 1. Following the analysis processes, CsTFs selection, co-expression classification from DEGs, promoter element scanning and motif enrichment analysis were applied to infer CsTF regulations.

### Condition specific transcription factors

**Fig. 2:**
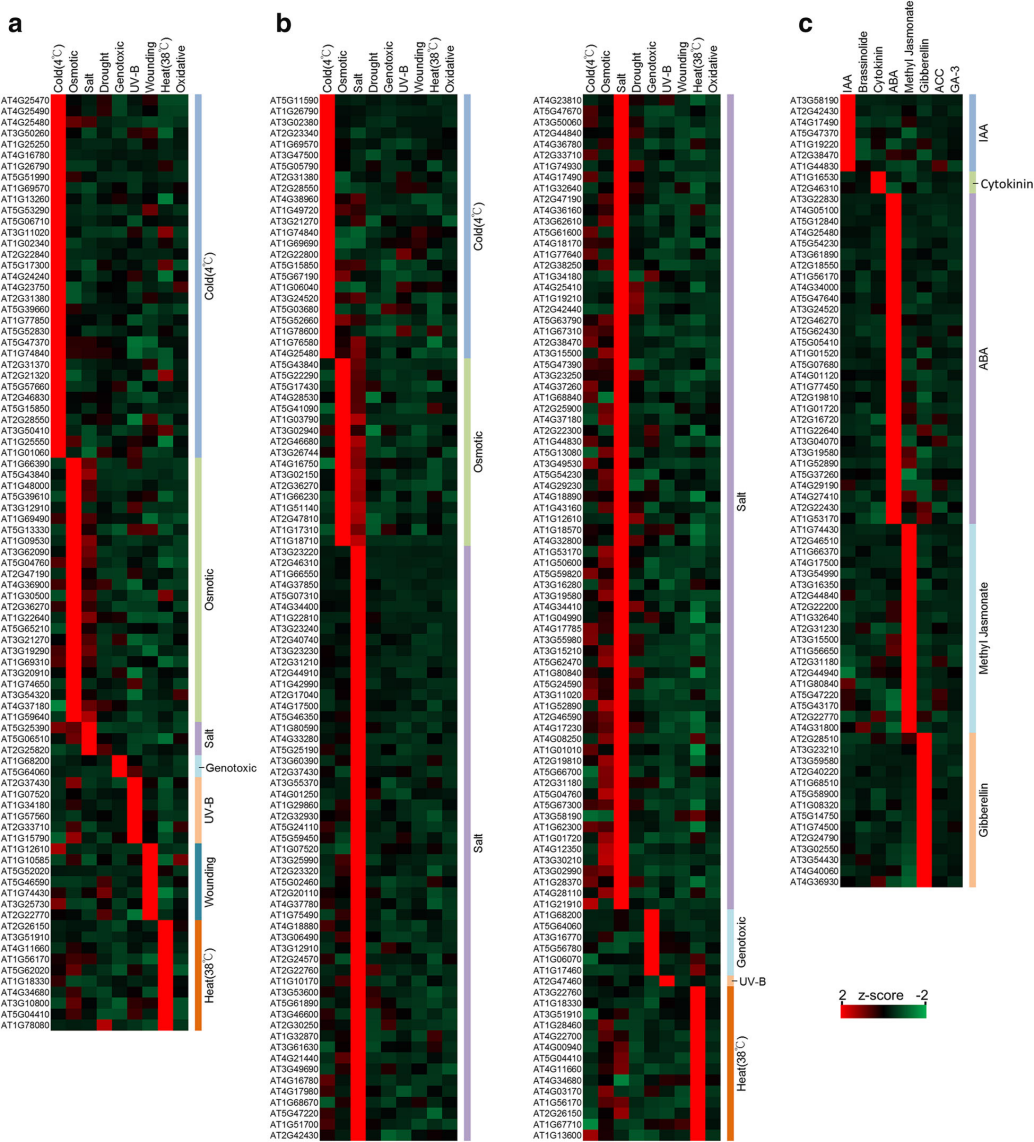
Expression pattern of CsTFs. The z-score profile indicated that each CsTF is strongly induced in one condition. CsTFs are ordered according to their condition clusters (marked by different colors in the right of heat maps). **a** and **b** show expression patterns of CsTFs under abiotic stresses in shoot and root, respectively. **c** shows expression patterns under eight hormone treatments

291 CsTFs in responses to specific abiotic stresses and hormone treatments were identified based on gene expression data (Fig. 2). All CsTFs under 26 conditions are listed in Additional file 2: Table S5. Several CsTFs are consistent with known essential regulators in their corresponding conditions. For example, under heat stress in shoot, four CsTFs are heat-shock transcription factor (HSF) family proteins which have reported as key factors in heat responsiveness. All of these, At2g26150 (Heat stress transcription factor A-2, HSFA2), AT3G51910 (Heat stress transcription factor A-7a, HSFA7A), AT4G11660 (Heat stress transcription factor B-2b, HSFB2B) and AT5G62020 (Heat stress transcription factor B-2a, HSFB2A), are essential regulatory components to mediate the heat tolerance pathway during heat shock [35, 36]. Moreover, three cold-specific TFs, AT4G25470 (C-repeat/DRE binding factor 2, CBF2), AT4G25490 (C-repeat/DRE binding factor 1, CBF1) and AT4G25480 (C-repeat/DRE binding factor 3, CBF3) have been reported to regulate over 100 genes when plants are exposed to low temperature [36, 37]. For ABA responsiveness, AT4G34000 (ABRE binding protein, ABF3) is a major transcription factor in plant ABA signalling transduction [38]. However, over half of CsTFs have little information about their regulatory mechanisms and DNA binding sites (summarized in Additional file 1: Table S6-S8). These CsTFs could be novel candidates for further experimental validation under specific conditions.

Numerous osmotic-specific TFs shown higher expression levels in salt stress than other conditions, and revealed strong correlation between salt and osmotic stress. This phenomenon was also identified in salt-specific TFs (Fig. 2). Several known and novel CsTFs related to cross talk between abiotic stresses and hormones were investigated. For instance, AT1G74430 (MYB95) and AT2G22770 (NAI1) are expected to regulate methyl jasmonate- and wounding-induced genes expression. This is consistent with the previous research which displayed that methyl jasmonate mediates plant responses against mechanical wounding cause by insects and pathogens [39, 40]. Moreover, NAI1 is a well-known regulator which affects the formation of ER body after plants are wounded [41, 42]. In addition, the correlations between methyl jasmonate and salt stress in root shows that three members (AT2G44840 (ERF13), AT4G17500 (ERF1A), and AT5G47220 (ERF2)) of ethylene response factor (ERF) family might play important roles in stress and hormone cross-talking.

### Motif preferences among co-expressed promoters (number and presence enrichment analysis)

The highest percentage of annotated TFs related to plant heat responsiveness was discovered in the heat-specific TF group, and more TF binding sites are known when comparing to other conditions (in Additional file 1: Table S6). Heat stress was thus selected as a case to examine the motif preferences of CsTFs. Table 1 summarizes the results for ten heat stress specific TFs (CsTFs under heat stress) belonging to six families. To investigate the transcriptional regulation mechanisms, 4–8-mer motif enrichments were evaluated on the promoters of co-expression genes for each CsTF. Due to the limited number of co-expressed genes, AT1G78080 (ethylene-responsive transcription factor RAP2.4) was discarded before further analysis.

**Table 1:** Summary of 10 CsTFs under heat stress in shoot

TF	co.G+ DEG1.5^a^	A^b^	Name	Family	Name of TFBS	Sequence of TFBS
AT1G18330	66	–	MYB-related transcription factor EPR1	Myb/SANT; MYB-related	–	–
AT1G56170	67	–	Nuclear transcription factor Y subunit C-2	NF-YC	–	CCAAT
AT1G78080	0	yes	Ethylene-responsive transcription factor RAP2–4	AP2; ERF	GCC-box pathogenesis-related promoter element	–
AT2G26150	72	yes	Heat stress transcription factor A-2	HSF	heat shock elements (HSE)	5’-AGAAnnTTCT-3’
AT3G10800	30	–	–	bZIP	–	–
AT3G51910	43	yes	Heat stress transcription factor A-7a	HSF	heat shock elements (HSE)	5’-AGAAnnTTCT-3’
AT4G11660	80	–	Heat stress transcription factor B-2b	HSF	heat shock elements (HSE)	5’-AGAAnnTTCT-3’
AT4G34680	67	yes	GATA transcription factor 3	GATA	–	5′-GATA-3′;5′-GAT-3’
AT5G04410	55	–	NAC domain-containing protein 78	NAC; NAM	–	–
AT5G62020	66	–	Heat stress transcription factor B-2a	HSF	heat shock elements (HSE)	5’-AGAAnnTTCT-3’

^a^the number of co-expressed genes (intersection of co-expressed genes (PCC ≥ 0.8) and DEGs under heat stress (p-value < 0.01; | log_2_ (FC) | ≥ 1.5))

^b^activator

**Fig. 3:**
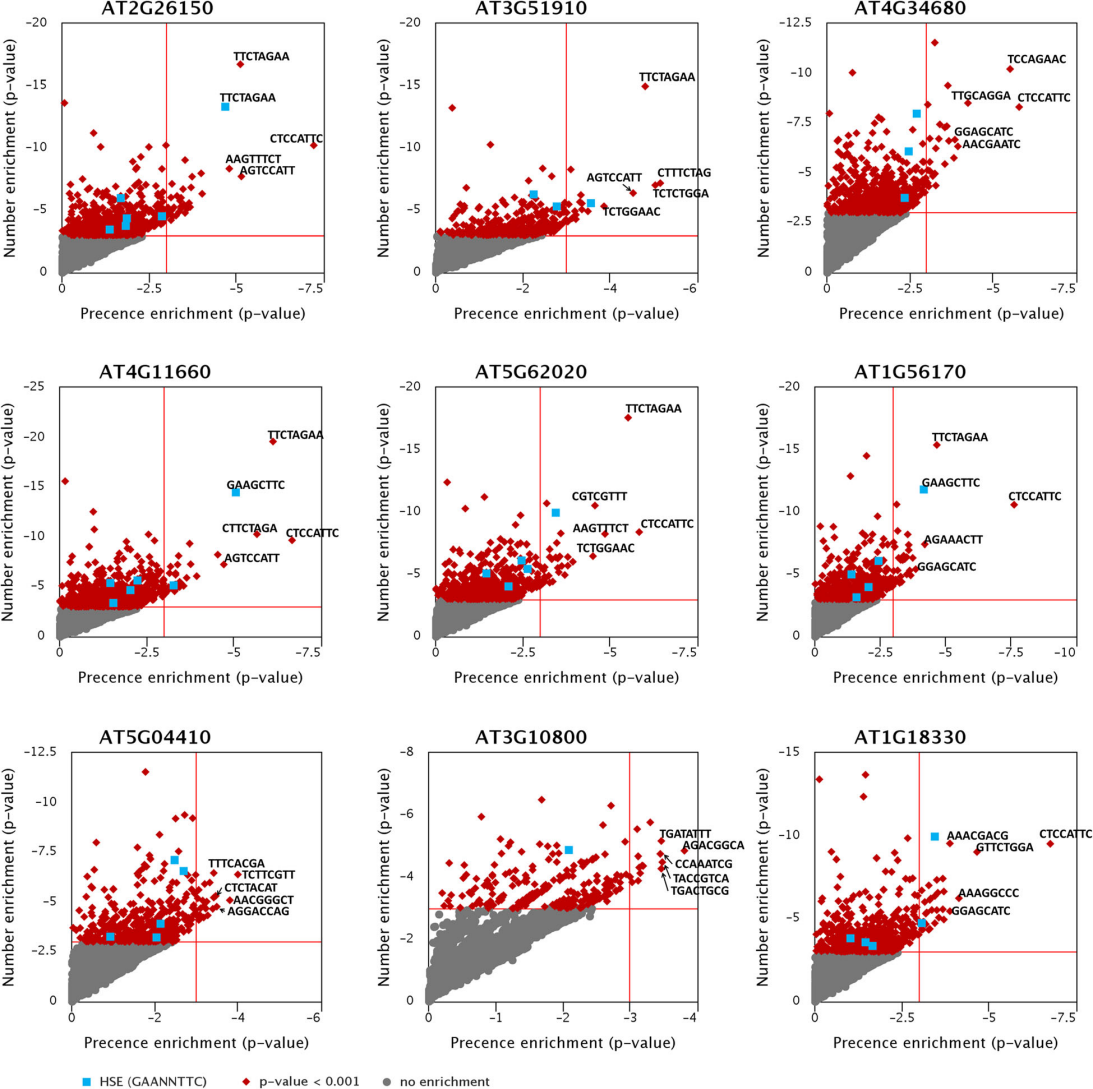
Scatter plots show *p*-values of presence and number enrichment for all observed 8-mer motifs. *P*-values are performed under log_10_ scale. Vertical and horizontal red lines are the thresholds of enrichment, 10e-3. Based on these two lines, each scatter plot can separate in four quadrants. Eight-mer motifs locating in first quadrant are defined as enriched motifs, which are both presence enrichment and number enrichment. The motifs in second quadrants are number enrichment instead of presence enrichment. The 8-mer sequences marked on sequences indicate the top five most-enriched motifs

The morphological and physiological alterations usually depends on the presence of stress-responsive *cis*-acting elements and their occurrence frequency on target promoters when plant adapt to environmental changes [43]. To set up a reasonable criterion that could distinguish actual *cis*-acting elements from promoters, we assessed the enrichment of the presence and number of 4–8-mer motifs among co-expressed genes promoters (see Additional file 1: Figure S3-S7). Interestingly, 8-mer motifs with presence enrichment also shown significant number enrichment (Fig. 3). The presence enriched motifs underwent number enrichment with markedly low *p*-values (under 10e-4). By contrast, the motifs lacking presence enrichment did not occupy high percentages of the co-expressed group compared with whole genome. In the case of four HSF proteins, even though more motifs fitting the heat shock element (HSE; GAANNTTC) were found in the second quadrant of scatter plots than the first quadrant, numerous motif in the second quadrant might be false positives (Fig. 3). These results indicate that if a motif is important for a CsTF, the significant presence of the motif seems to be required in the promoters of the co-expressed genes. The presence enrichment thus is a good index to evaluate the important of motifs for a CsTF. Based on above, such presence and number enrichments show the applicability to select motifs from promoter sequences.

### Enriched motifs reveal characteristic of actual stress responsive regulations

A conserved repetitive palindromic motif (GAAnnTTC) is a well-known HSE in upstream promoters of heat-inducible genes. Previous studies also indicated that the recognition of HSE is required for the regulation of HSFs [44, 45]. Therefore, we are interested in whether palindromic elements are significant in the enriched 8-mer motifs. Among the enriched 8-mer motifs of seven heat stress specific TFs, at least three types of palindromic motifs have been found (in Additional file 1: Table S9). Interestingly, most of these were (GAAnnTTC), a typical HSE, or (TTCnnGAA) which exchanged the order of two 3-mer core sequence of HSE. This palindromic sequence also indicates that the heat stress specific TFs might function as a dimer. The structure of the HSF-DNA complex from *Kluyveromyces lactis* (PDB Id: 3HTS) also illustrates the same issue (Additional file 1: Figure S8) [46].

The members from the same TF family which were classified based on similar DNA binding domains tend to display similar DNA binding specificity [13, 14, 47, 48]. The position specific scoring matrices for six families of heat stress specific TFs (bZIP, NAC; NAM, Myb/SANT; MYB-related, GATA, NF-YC, HSF) from PlantPAN 2.0 show that most of the matrices for the same TF family were similar to each other (in Additional file 1: Table S10) [28]. These matrices also illustrated that the conserved core sequences for half of heat stress responsive families may prefer to be 3-mer motifs (Additional file 1: Figure S9A). To examine the properties observed in experimental binding sites, we investigated the occurrences of 3-mer in all enriched 8-mer for 9 CsTFs. The top 10 significant entries revealed that a sequence (GAA) was common in four HSF proteins and was highly overrepresented (in Additional file 2: Table S2). In addition, the other two sequences (TTC) and (TCT) were also highly presented in the enriched 8-mer in HSF proteins. For other CsTFs (AT1G18330, AT5G04410, AT4G34680, and AT1G56170), the sequence (GAA) shown higher frequency than the other 3-mer motifs. This reveals that GAA motif is a vital *cis*-acting element for gene transcriptional regulation during heat stress responsiveness. Taken together, the palindromic feature and the heat-responsive overrepresented 3-mer demonstrate that our bioinformatics approach could successfully identify critical *cis*-acting elements.

### Determination of DNA binding sequences for CsTFs

**Fig. 4:**
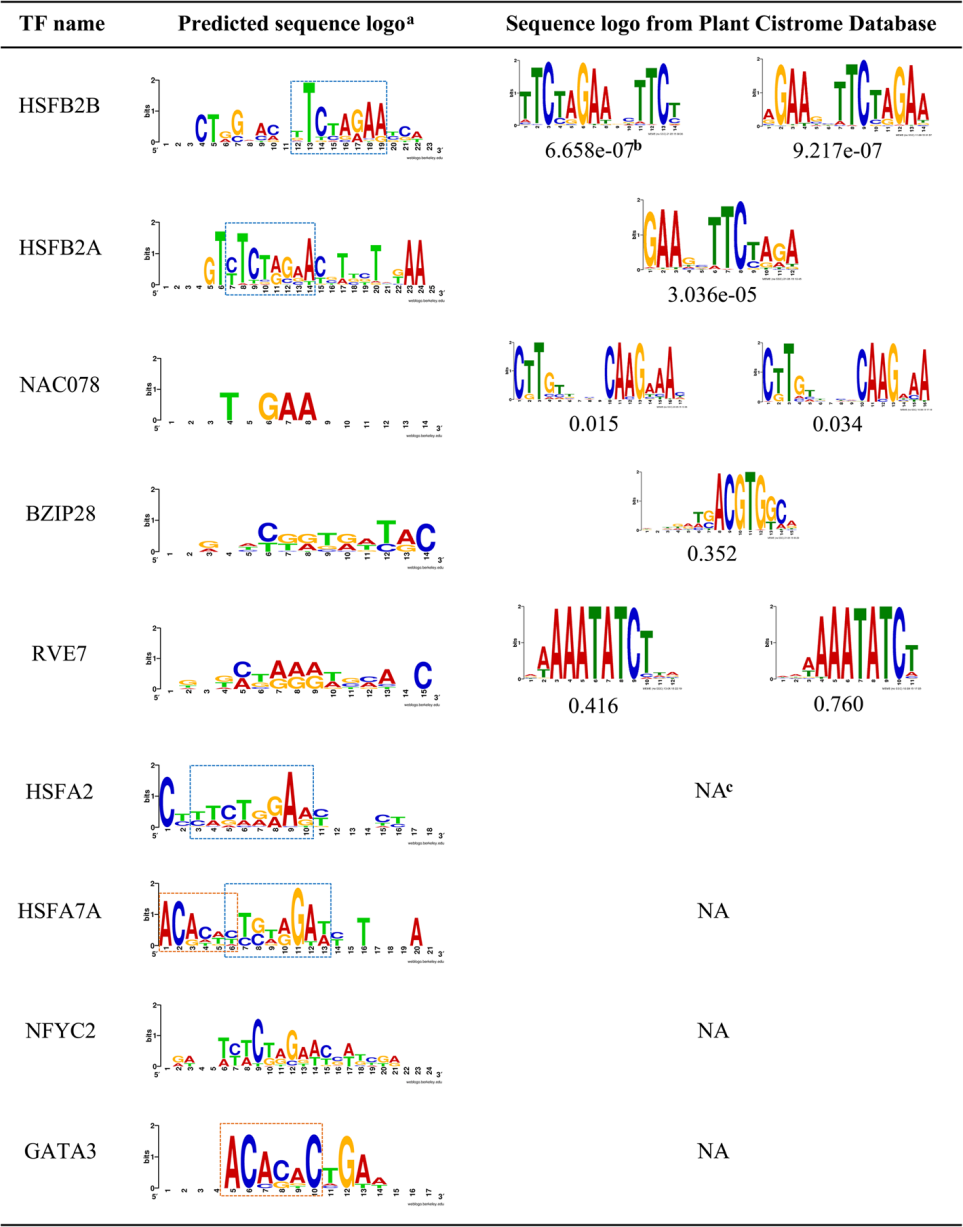
Sequence logos of CsTFs under heat stress. ^a^:HSEs (GAANNTTC) are marked in blue dotted rectangles. AC-rich flanking sequences are mark in orange dotted rectangles. ^b^: motif-motif similarity (*P*-value) is calculated by Tomtom. ^c^: No sequence logo could be obtained from Plant Cistrome Database

The interaction preferences among TFs and target genes are crucial for transcriptional regulation. To reveal the potential DNA binding sites for a CsTF, the position specific scoring matrices were generated according to the occurrence and overlapping of the enriched 8-mer motifs in the promoters. In co-expressed gene promoters, the overlapping motifs are enriched particularly within ~ 500 bp upstream of the transcriptional start site (Additional file 1: Figure S10). This position bias of the overlapping motifs was similar to the experimentally verified motifs in plants [49, 50]. The sequence logos for each CsTF are displayed in Fig. 4. Motif comparisons of eight matrices from five heat stress specific TFs show that three matrices corresponding to HSFB2B and HSFB2A are similar to those from the published database (Fig. 4) [16]. For the four HSF proteins, their sequences present the perfect type HSE (GAANNTTC). Among these, HSFA7A was slightly different from the others, with additional AC-rich flanking sequences at its 5’end of the sequence logo. Interestingly, a member of the GATA family, AT4G34680 (GATA transcription factor 3, GATA3) also preferred to recruit this novel motif in the promoters of its co-expressed genes.

### A case study ABA-CsTF identification

To further demonstrate the applications of our approach to hormone treatment, the CsTFs for ABA treatment were taken as an example, due to more known motifs can be referred (in Additional file 1: Table S8). Following the analysis procedure, 30 CsTFs were identified as ABA specific regulators belonging to 16 families (in Additional file 2: Table S5). According to the confirmed binding matrixes of TF families, the significant 4-mer in the enriched 8-mer motif might be efficient sequences to recognize the essential *cis*-acting elements for a specific condition (Additional file 1: Figure S9). Notably, by assessing the overrepresented 4-mer in enriched 8-mer motifs, a sequence (ACGT), the core flanking of ABRE, is recognized in all ABA-CsTFs (in Additional file 2: Table S3). An “ACGT” is a top one overrepresented 4-mer sequence identified in 80% of ABA-CsTFs, and shows within top five 4-mer sequence in the other 20%. This suggests that the presence of the ACGT motif in gene promoters is necessary for ABA responsive regulation in *Arabidopsis*, which is consistent with current studies [51, 52].

To further validate the candidate *cis*-elements under ABA treatment, the developed methods were employed in *Oryza sativa*. The microarray expressions data from six hormones treatments (ABA, auxin, brassinosteroid, cytokinin, gibberellin, and jasmonic acid) (in Additional file 1: Table S4) were used. Following our analytical methods, 60 and 69 rice ABA-CsTFs were identified from shoot and root, respectively. Expectedly, a well-known ABRE core motif (ACGT) were investigated in top ten overrepresented 4-mer of ABA-CsTF in rice. Interestingly, the top ten overrepresented 4-mer were slightly different when comparing between rice and *Arabidopsis* (Additional file 1: Figure S11). Although “ACGT” was identified in the top ten overrepresented 4-mer for rice ABA-CsTFs, the higher percentage of “CACG” and “CCAC” were recognized and might be novel *cis*-elements for ABA responsiveness. The 3′ and 5′ flank sequences of ABRE core motif (ACGT) was different between *Arabidopsis* (AC**ACGT**GTC) and rice (CC**ACGT**), which suggests the specificity of species. Except for known ABRE, the other top ten overrepresented 4-mer also performed various preference between *Arabidopsis* and rice (Additional file 1: Figure S11). For example, “GTGG”, “AATA”, and “TACG” were especially for *Arabidopsis*, but not for rice. Together, the rice ABA-CsTFs and ABRE core motifs suggest that our approach can be widely applied to explore CsTFs and condition specific *cis*-elements in plant. The rice/*Arabidopsis* comparisons also demonstrate that the variance among species can also be retrieved in CsTF analysis.

**Fig. 5:**
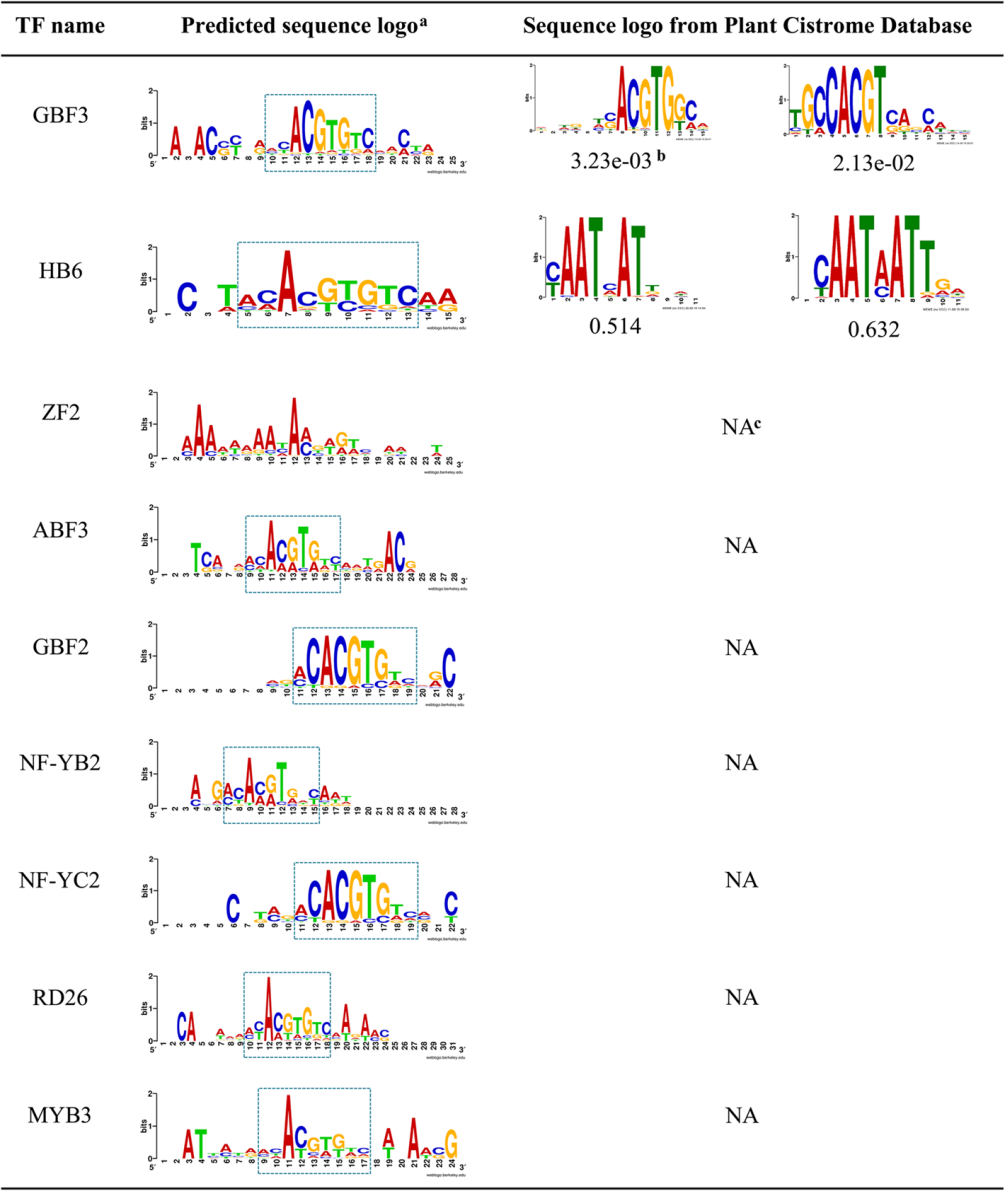
Sequence logos of nine CsTFs under ABA treatment. ^a^: G-box motifs (ACACGTGTC) are marked in dotted rectangles. ^b^: motif-motif similarity (*P*-value) calculated by Tomtom. ^c^: No sequence logo could be obtained from Plant Cistrome Database

With regard to the potential DNA binding sites for an individual CsTF, the sequence logos of *Arabidopsis* ABA-CsTFs were demonstrated in Fig. 5. Significantly, the sequence logos of AT4G34000 (ABA responsive elements-binding factor 3, ABF3), which has been experimentally confirmed to regulate the ABA signalling pathway, was consistently matched to the preference of known binding sites (ACACGTGT). Except for AT3G19580 (zinc-finger protein 2, ZF2), other eight ABA-CsTFs, bind to G-box motifs, which have been verified by ChIP-seq (Fig. 5) [15]. In summary, the results of ABA and heat stress analysis illustrated that our approach is suitable to explore and uncover the *cis*-acting regulation for abiotic stresses and hormone treatments. Information on all other conditions can be retrieved from our online database (http://wcchang.itps.ncku.edu.tw/CsTFAnalysis/).

## Discussion

In this study, we developed a series of computational methods to discover specific *trans*- and *cis-* regulatory elements under a particular condition. Among 291 CsTFs, several of these were confirmed with regard to their corresponding conditions (in Additional file 2: Table S5). Overlapping the CsTF list between root and shoot, we observed that 37% of the overlapping CsTFs act as salt-responsive regulators in root while they were induced due to different abiotic stresses in shoot. This may illustrates that the plant root needs more direct and specific regulations to resist salt stress than shoot. The other possibility is that shoot response to salt stress is indirect and affected by second messengers. More protein partners or co-factors might be required for TFs in shoot to co-regulate their target genes under salt stress. Additionally, the overlapping between abiotic stresses and hormone treatment shows that plant resistant abiotic stress is usually related to endogenous stimulus.

Although more advanced computational approaches and experiments have been developed to investigate stress-responsive TFs and their regulatory networks, the relationships between TFs and their binding sites remain unknown. The results of sequence logos demonstrate that our method can successfully generate potential binding sites for CsTFs. In the cases of heat stress specific TFs, GO enrichment analysis of the co-expressed genes reveals that the four HSF proteins show significant enrichment in major functions, such as response to heat and response to stress (in Additional file 1: Table S11). However, they play different roles in several sub-functions (in Additional file 1: Table S12). These differences thus demonstrate the complexity of heat stress regulation, since some of the HSF proteins generally cooperate with other TFs to activate distinct gene functions in plant cells [44, 53]. In the overlapping CsTFs list, with regard to responses to heat stress in shoot and root, we found that, in root, heat stress-TFs tend to bind promoter elements which contain three repetitive core sequences (GAA) of HSE compared to only two repetitions in shoot (data not shown). This finding suggests that the integration of condition-specific *cis*-elements under various conditions can provide further characteristics to distinguish TF binding sites in distinct plant organs (tissues).

To examine the reliability of the results, we compared the ABA specific DNA-binding sequences from our computational approaches to those from in vivo experiments. Among 10 ABA-CsTF which have been reported as ABA-related TFs in the previous study, the sequence logos of eight ABA-CsTF show the same preferences (A**CACGTG**TC) of ChIP-seq experiments [15]. They contain a core sequence of G-box motif (CACGTG) and two flanking sequence, A- and –TC at 5′ and 3′ sides, respectively. This finding shows our bioinformatics approach for genome-wide promoter analysis can be used to infer valuable *cis-*acting regulation in response to a stimulus with high consistent with the in vivo verified motifs. Though we demonstrated the application of our approach to abiotic stress and hormone treatments, it may be useful for studying the relationships among TFs and *cis-*acting elements using other gene expression data, such as RNA-sequencing or derived conditions, e.g. developmental stages and biotic stresses.

## Conclusions

This study provides new bioinformatics approach combining microarray expression data and genomic promoter sequences for identification of condition specific *cis*- and *trans*- regulatory elements. Several known and novel *cis*-acting elements were identified for 291 CsTFs and 26 conditions. The results of heat stress and ABA treatment suggest that overrepresented 3- and 4-mer motifs in an enriched 8-mer motif could be a core *cis*-element for a CsTF. The overall results illustrate this study may be useful in identifying condition specific *cis*- and *trans*- regulatory elements and facilitate our understanding of the relationships among TFs, *cis-*acting elements and target gene expression.

## Additional files

Additional file 1: Tables S1, S4, and S6 to S12. Figures S1 to S11. Supplementary Tables and Figures. (DOC 4939 kb)

Additional file 2: Tables S2, S3, and S5. Top 10 significant entries of overrepresented 3-mer and 4-mer sequences for heat stress specific TFs and ABA-CsTFs, respectively, and CsTFs lists in three condition datasets. (XLS 143 kb)

## Abbreviations

ChIP-chip: Chromatin immunoprecipitation combining with microarray
ChIP-seq: Chromatin immunoprecipitation combining with sequencing
CsTF: Condition specific transcription factor
DAP-seq: DNA affinity purification sequencing
DEG: Differentially expressed gene
DFTF: Differentially expressed TF
ERF: Ethylene response factor
HSE: Heat shock element
HSF: Heat-shock transcription factor
PCC: Pearson correlation coefficients
TF: Transcription factor
