# Supplementary material for: Delineation of condition specific Cis- and Trans-acting elements in plant promoters under various Endo- and exogenous stimuli
Source: BMC Genomics. 2018 May 9;19(Suppl 2):85. doi: 10.1186/s12864-018-4469-4 (PMC5954277; doi:10.1186/s12864-018-4469-4)
Supplement: Supplementary file 1 — Supplementary Tables and Figures. (DOC 4939 kb) [file 12864_2018_4469_MOESM1_ESM.doc]

**Additional file Tables and Figures**

**Table S1. Three datasets of microarray samples for *Arabidopsis*.**

| **Dataset** | **Stresses** | | **# of samples** |
| --- | --- | --- | --- |
|  | **Treatment** | **control** |  |
| Abiotic stress in  root | Cold stress (30m, 1h, 3h, 6h, 12h, 24h) | Control (30m, 1h, 3h, 6h, 12h, 24h) | 124 |
|  | Drought stress (15m, 30m, 1h, 3h, 6h, 12h, 24h) | Control (15m, 30m, 1h, 3h, 6h, 12h, 24h) |  |
|  | Genotoxic stress (30m, 1h, 3h, 6h, 12h, 24h) | Control (30m, 1h, 3h, 6h, 12h, 24h) |  |
|  | Heat stress (15m, 30m, 1h, 3h) | Control (15m, 30m, 1h, 3h) |  |
|  | Osmotic stress (30m, 1h, 3h, 6h, 12h, 24h) | Control (30m, 1h, 3h, 6h, 12h, 24h) |  |
|  | Oxidative stress (30m, 1h, 3h, 6h, 12h, 24h) | Control (30m, 1h, 3h, 6h, 12h, 24h) |  |
|  | Salt stress (30m, 1h, 3h, 6h, 12h, 24h) | Control (30m, 1h, 3h, 6h, 12h, 24h) |  |
|  | UV-B stress (15m, 30m, 1h, 3h, 6h, 12h, 24h) | Control (15m, 30m, 1h, 3h, 6h, 12h, 24h) |  |
|  | Wounding stress (15m, 30m, 1h, 3h, 6h, 12h, 24h) | Control (15m, 30m, 1h, 3h, 6h, 12h, 24h) |  |
| Abiotic stress in shoot | Cold stress (30m, 1h, 3h, 6h, 12h, 24h) | Control (30m, 1h, 3h, 6h, 12h, 24h) | 124 |
|  | Drought stress (15m, 30m, 1h, 3h, 6h, 12h, 24h) | Control (15m, 30m, 1h, 3h, 6h, 12h, 24h) |  |
|  | Genotoxic stress (30m, 1h, 3h, 6h, 12h, 24h) | Control (30m, 1h, 3h, 6h, 12h, 24h) |  |
|  | Heat stress (15m, 30m, 1h, 3h) | Control (15m, 30m, 1h, 3h) |  |
|  | Osmotic stress (30m, 1h, 3h, 6h, 12h, 24h) | Control (30m, 1h, 3h, 6h, 12h, 24h) |  |
|  | Oxidative stress (30m, 1h, 3h, 6h, 12h, 24h) | Control (30m, 1h, 3h, 6h, 12h, 24h) |  |
|  | Salt stress (30m, 1h, 3h, 6h, 12h, 24h) | Control (30m, 1h, 3h, 6h, 12h, 24h) |  |
|  | UV-B stress (15m, 30m, 1h, 3h, 6h, 12h, 24h) | Control (15m, 30m, 1h, 3h, 6h, 12h, 24h) |  |
|  | Wounding stress (15m, 30m, 1h, 3h, 6h, 12h, 24h) | Control (15m, 30m, 1h, 3h, 6h, 12h, 24h) |  |
| Hormones | ABA (30m, 1h, 3h) | Control (30m, 1h, 3h) | 96 |
|  | Auxin (30m, 1h, 3h) | Control (30m, 1h, 3h) |  |
|  | Brassinolide (30m, 1h, 3h) | Control (30m, 1h, 3h) |  |
|  | Brassinosteroids (30m, 1h, 3h) | Control (30m, 1h, 3h) |  |
|  | GA (30m, 1h, 3h) | Control (30m, 1h, 3h) |  |
|  | Gibberellin (3h, 6h, 9h) | Control (3h, 6h, 9h) |  |
|  | Jasmonic acid (30m, 1h, 3h) | Control (30m, 1h, 3h) |  |
|  | Zeatin ( cytokinin ) (30m, 1h, 3h) | Control (30m, 1h, 3h) |  |

**Table S4.** Two datasets of microarray samples for rice.

| **Dataset** | **Stresses** | | **# of samples** |
| --- | --- | --- | --- |
|  | **Treatment** | **control** |  |
| Hormones in root | Abscisic acid (15m, 30m, 1h, 3h, 6h) | Control (15m, 30m, 1h, 3h, 6h) | 180 |
|  | Auxin (15m, 30m, 1h, 3h, 6h) | Control (15m, 30m, 1h, 3h, 6h) |  |
|  | Brassinosteroid (15m, 30m, 1h, 3h, 6h) | Control (15m, 30m, 1h, 3h, 6h) |  |
|  | Cytokinin (15m, 30m, 1h, 3h, 6h) | Control (15m, 30m, 1h, 3h, 6h) |  |
|  | Gibberellin (15m, 30m, 1h, 3h, 6h) | Control (15m, 30m, 1h, 3h, 6h) |  |
|  | Jasmonic acid (15m, 30m, 1h, 3h, 6h) | Control (15m, 30m, 1h, 3h, 6h) |  |
| Hormones in shoot | Abscisic acid (1h, 3h, 6h, 12h) | Control (1h, 3h, 6h, 12h) | 96 |
|  | Auxin (1h, 3h, 6h, 12h) | Control (1h, 3h, 6h, 12h) |  |
|  | Brassinosteroid (1h, 3h, 6h, 12h) | Control (1h, 3h, 6h, 12h) |  |
|  | Cytokinin (1h, 3h, 6h, 12h) | Control (1h, 3h, 6h, 12h) |  |
|  | Gibberellin (1h, 3h, 6h, 12h) | Control (1h, 3h, 6h, 12h) |  |
|  | Jasmonic acid (1h, 3h, 6h, 12h) | Control (1h, 3h, 6h, 12h) |  |

**Table S6.** Statistics of CsTFs under seven abiotic stresses in shoot.

|  | **Number of CsTFs** | | |
| --- | --- | --- | --- |
| **Conditions** | **total** | **respond to this condition^a^** | **with known motif^b^** |
| Cold (4℃) | 33 | 7 | 14 |
| Osmotic | 24 | 3 | 7 |
| Salt | 3 | 2 | 1 |
| Genotoxic | 2 | 0 | 0 |
| UV-B | 6 | 2 | 0 |
| Wounding | 7 | 2 | 1 |
| Heat (38℃) | 10 | 5 | 6 |

^a^: the number of CsTFs which have been reported to respond to the condition described in this study.

^b^: the number of CsTFs with known corresponding binding sites (TFBSs).

**Table S7. Statistics of CsTFs under seven abiotic stresses in root.**

|  | **Number of CsTFs** | | |
| --- | --- | --- | --- |
| **Conditions** | **total** | **respond to this condition^a^** | **with known motif^b^** |
| Cold(4℃) | 24 | 4 | 7 |
| Osmotic | 17 | 1 | 4 |
| Salt | 128 | 24 | 29 |
| Genotoxic | 6 | 0 | 0 |
| UV-B | 1 | 0 | 0 |
| Wounding | 0 | - | - |
| Heat(38℃) | 14 | 3 | 7 |

^a^: the number of CsTFs which have been reported to respond to the condition described in this study.

^b^: the number of CsTFs with known corresponding binding sites (TFBSs).

**Table S8.** Statistics of CsTFs under eight hormone treatments.

|  | **Number of CsTFs** | | |
| --- | --- | --- | --- |
| **Conditions** | **total** | **respond to this condition^a^** | **with known motif^b^** |
| IAA | 7 | 2 | 2 |
| Cytokinin | 2 | 1 | 0 |
| ABA | 30 | 5 | 11 |
| JA | 19 | 1 | 4 |
| Gibberellin | 14 | 1 | 3 |
| ACC | 0 | - | - |
| GA-3 | 0 | - | - |
| Brassinolide | 0 | - | - |

^a^: the number of CsTFs which have been reported to respond to the condition described in this study.

^b^: the number of CsTFs with known corresponding binding sites (TFBSs).

**Table S9.** Palindromic motifs in enriched 8-mer motifs for CsTFs under heat stress.

| **TF** | **Palindromic motifs** |
| --- | --- |
| AT2G26150 | GAAGCTTC |
|  | TTCTAGAA |
|  | TTCTGGAA |
|  | GAATCTTC |
| AT3G51910 | GAAGTTTC |
|  | GAATCTTC |
|  | TTCTAGAA |
| AT4G11660 | GAACCTTC |
|  | TTCTAGAA |
|  | GAAGCTTC |
|  | TTCTGGAA |
| AT5G62020 | TTCTAGAA |
|  | CGACGTCG |
|  | GAAGCTTC |
|  | TTCTGGAA |
|  | GAATCTTC |
| AT1G18330 | GAAGGTTC |
|  | GAACCTTC |
|  | GAAGCTTC |
|  | CCTTTAGG |
|  | CCTAAAGG |
| AT1G56170 | TTCTAGAA |
|  | CGACGTCG |
|  | GAAGCTTC |
|  | TTCCAGAA |
|  | TTCTGGAA |
| AT4G34680 | TTCCAGAA |
|  | TTCTGGAA |
|  | TTCTAGAA |

**Table S10.** The matrixes of six CsTF families under heat stress.

| Family | Matrixes from PlantPAN2.0 |
| --- | --- |
| GATA | TF_motif_seq_0237, TF_motif_seq_0243, TFmatrixID_0259, TFmatrixID_0260, TFmatrixID_0261, TFmatrixID_0262, TFmatrixID_0263, TFmatrixID_0264, TFmatrixID_0265, TFmatrixID_0266, TFmatrixID_0267, TFmatrixID_0268, TFmatrixID_0269, TFmatrixID_0270, TFmatrixID_0271, TFmatrixID_0272, TFmatrixID_0273 |
| bZIP | TF_motif_seq_0014, TF_motif_seq_0019, TF_motif_seq_0082, TF_motif_seq_0088, TF_motif_seq_0220, TF_motif_seq_0240, TF_motif_seq_0271, TF_motif_seq_0281, TF_motif_seq_0287, TF_motif_seq_0424, TF_motif_seq_0451, TFmatrixID_0027, TFmatrixID_0028, TFmatrixID_0181, TFmatrixID_0182, TFmatrixID_0183, TFmatrixID_0184, TFmatrixID_0185, TFmatrixID_0186, TFmatrixID_0187, TFmatrixID_0188, TFmatrixID_0189, TFmatrixID_0190, TFmatrixID_0191, TFmatrixID_0192, TFmatrixID_0193, TFmatrixID_0194, TFmatrixID_0195, TFmatrixID_0196, TFmatrixID_0197, TFmatrixID_0198, TFmatrixID_0199, TFmatrixID_0202, TFmatrixID_0203, TFmatrixID_0483, TFmatrixID_0489, TFmatrixID_0490, TFmatrixID_0496, TFmatrixID_0515, TFmatrixID_0533, TFmatrixID_0545, TFmatrixID_0546, TFmatrixID_0619, TFmatrixID_0620 |
| NAC; NAM | TF_motif_seq_0305, TFmatrixID_0010, TFmatrixID_0011, TFmatrixID_0012, TFmatrixID_0013, TFmatrixID_0382, TFmatrixID_0388, TFmatrixID_0389, TFmatrixID_0390, TFmatrixID_0391, TFmatrixID_0392, TFmatrixID_0393, TFmatrixID_0394, TFmatrixID_0395, TFmatrixID_0396, TFmatrixID_0397, TFmatrixID_0381, TFmatrixID_0383, TFmatrixID_0384, TFmatrixID_0385, TFmatrixID_0386, TFmatrixID_0387 |
| NF-YC | TF_motif_seq_0257, TF_motif_seq_0367 |
| Myb/SANT; MYB-related | TFmatrixID_0320, TFmatrixID_0334, TFmatrixID_0363, TFmatrixID_0364, TFmatrixID_0369 |
| HSF | TF_motif_seq_0010, TFmatrixID_0045, TFmatrixID_0046, TFmatrixID_0641, TFmatrixID_0642 |

**Table S11.** The major function of four HSF under heat stress.

| **GO ID** | **GO term** | **AT2G26150** | **AT3G51910** | **AT4G11660** | **AT5G62020** |
| --- | --- | --- | --- | --- | --- |
| GO:0009644 | response to high light intensity | 1.70E-54 | 8.55E-39 | 4.34E-57 | 1.12E-60 |
| GO:0042542 | response to hydrogen peroxide | 1.06E-53 | 9.86E-40 | 3.19E-54 | 3.15E-55 |
| GO:0009408 | response to heat | 3.35E-52 | 1.62E-39 | 1.46E-52 | 5.35E-56 |
| GO:0006457 | protein folding | 2.69E-48 | 6.35E-34 | 1.20E-50 | 9.82E-48 |
| GO:0034976 | response to endoplasmic reticulum stress | 5.68E-28 | 4.01E-19 | 6.18E-29 | 9.28E-29 |
| GO:0006950 | response to stress | 1.24E-15 | 7.44E-12 | 1.54E-15 | 2.22E-16 |
| GO:0051082 | unfolded protein binding | 1.33E-06 | 5.75E-03 | 6.10E-08 | 2.91E-05 |
| GO:0010286 | heat acclimation | 1.53E-06 | 5.58E-06 | 2.34E-06 | 1.03E-06 |
| GO:0005622 | intracellular | 1.36E-05 | 4.96E-03 | 1.89E-06 | 1.90E-05 |
| GO:0005634 | nucleus | 3.93E-04 | 3.40E-03 | 2.18E-05 | 1.16E-04 |
| GO:0043565 | sequence-specific DNA binding | 5.99E-04 | 3.67E-04 | 1.37E-04 | 4.80E-05 |
| GO:0019538 | protein metabolic process | 9.06E-04 | 3.23E-04 | 1.07E-03 | 7.77E-04 |
| GO:0034605 | cellular response to heat | 1.26E-03 | 4.50E-04 | 1.49E-03 | 1.08E-03 |

**Table S12.** The sub-function of four HSF under heat stress.

| **GO ID** | **GO term** | **AT2G26150** | **AT3G51910** | **AT4G11660** | **AT5G62020** |
| --- | --- | --- | --- | --- | --- |
| GO:0005623 | cell | 1.15E-04 | 0.02 | 2.58E-05 | 1.23E-04 |
| GO:0005737 | cytoplasm | 2.10E-04 | 0.03 | 4.44E-05 | 7.82E-05 |
| GO:0005575 | Cellular component | 6.64E-03 | 0.05 | 1.49E-03 | 4.70E-03 |
| GO:0070370 | cellular heat acclimation | 1.02E-04 | NA^a^ | 1.21E-04 | 8.72E-05 |
| GO:0005528 | FK506 binding | 1.67E-03 | NA | 1.98E-03 | 1.43E-03 |
| GO:0018208 | peptidyl-proline modification | 1.67E-03 | NA | 1.98E-03 | 1.43E-03 |
| GO:0006499 | N-terminal protein myristoylation | 3.33E-03 | NA | 4.24E-03 | 2.67E-03 |
| GO:0046686 | response to cadmium ion | 6.14E-03 | 1.50E-01 | 1.68E-03 | 0.02 |
| GO:0043226 | organelle | 8.39E-03 | 0.02 | 3.15E-03 | 0.01 |
| GO:0000413 | protein peptidyl-prolyl isomerization | 9.94E-03 | NA | 0.01 | 8.56E-03 |
| GO:0003755 | peptidyl-prolyl *cis*-trans isomerase activity | 0.01 | NA | 0.01 | 9.15E-03 |
| GO:0005829 | cytosol | 0.03 | NA | 6.33E-03 | 5.57E-03 |
| GO:0009615 | response to virus | NA | NA | 2.93E-03 | 2.12E-03 |
| GO:0045087 | innate immune response | 0.03 | NA | 3.83E-03 | 0.03 |
| GO:0045893 | positive regulation of transcription, DNA-templated | NA | NA | 1.46E-03 | 0.02 |
| GO:0009816 | defense response to bacterium, incompatible interaction | NA | NA | 4.05E-03 | NA |

^a^: p-value of GO enrichment is larger than 0.01.


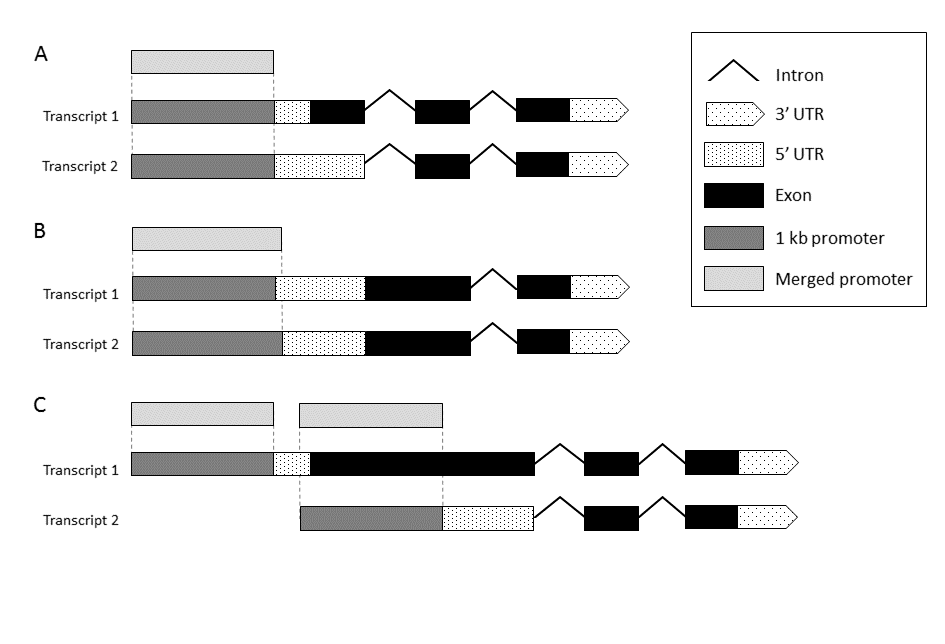


**Figure S1.** Three types of merged promoters between two transcripts. A gene with multiple transcripts were merged with regard to their promoters according their locations in the genome. The merged promoters include: (A) totally merged 1kb promoter, which appeared in two transcripts with the same transcriptional start sites, (B) extended promoter in two transcripts with different transcriptional start sites whose distance is shorter than 1kb, (C) two separated promoters. A gene with three or more transcripts that contain more than one type of merged promoter.


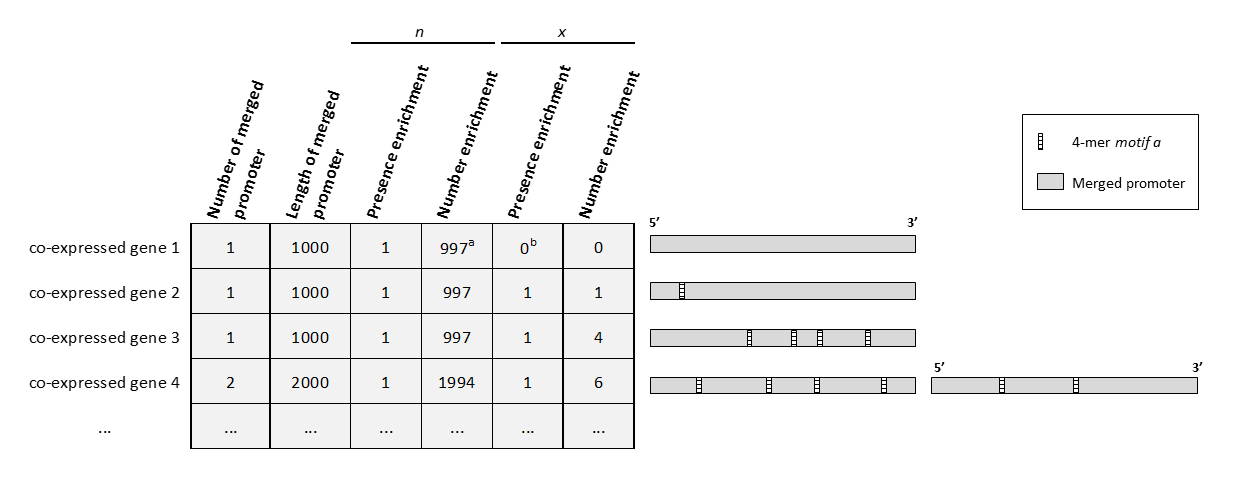


**Figure S2.** An example for calculating presence enrichment and number enrichment. A 4-mer *motif a* is used in this example. The rows represent all co-expressed genes of a CsTF. ^a^: how many possible 4-mer motifs are found in this co-expressed gene promoter. The sum of this column is *n* of number enrichment. ^b^: zero represents that *motif a* is not found in a co-expressed gene promoter. If a promoter contains *motif a*, this value will be marked as one, no matter how many *motif a* are in this promoter. The sum of this column is the *x* of presence enrichment.


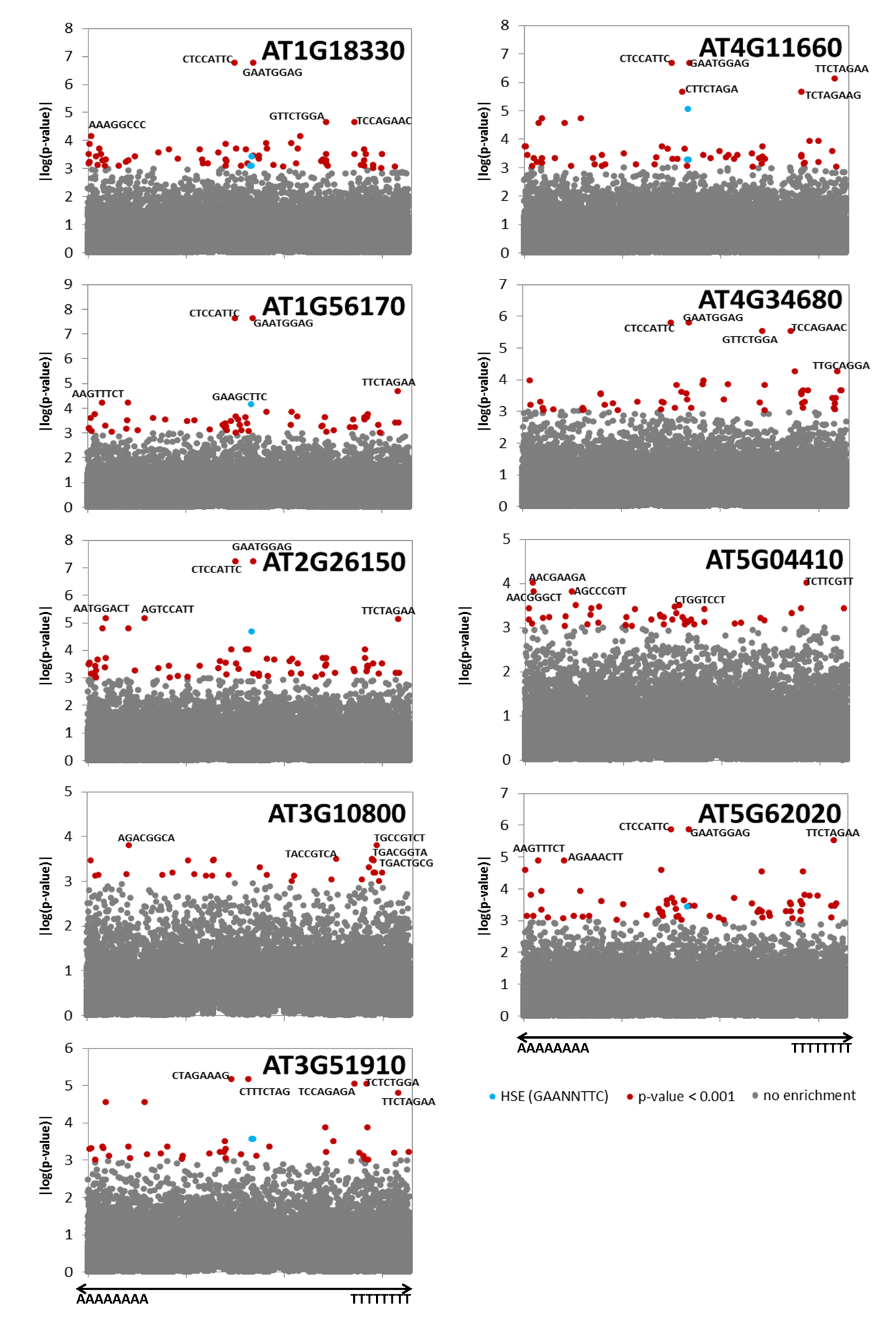


**Figure S3.** All possible 8-mer in the promoters of the co-expressed group for CsTFs under heat stress.


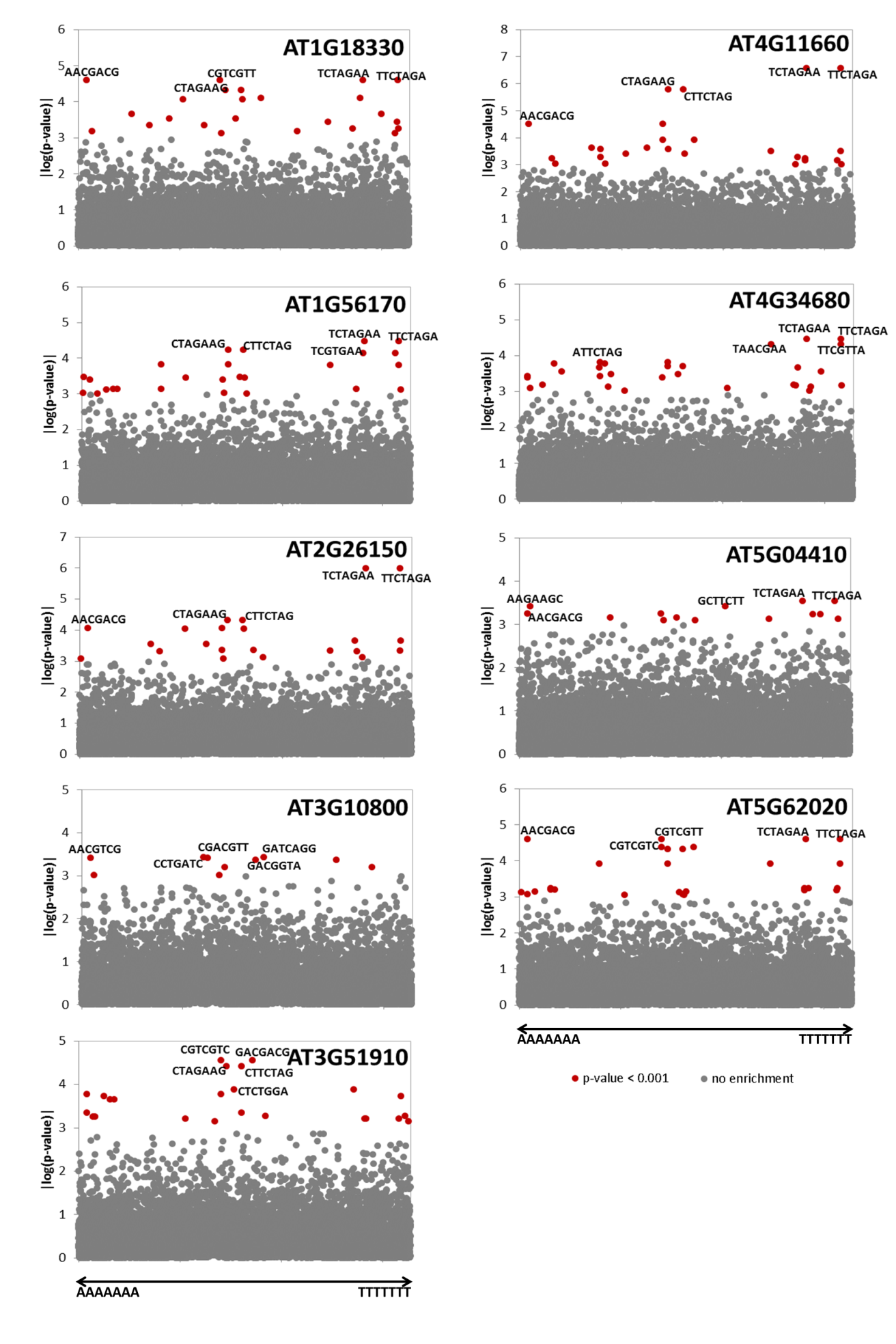


**Figure S4.** All possible 7-mer in the promoters of the co-expressed group for CsTFs under heat stress.


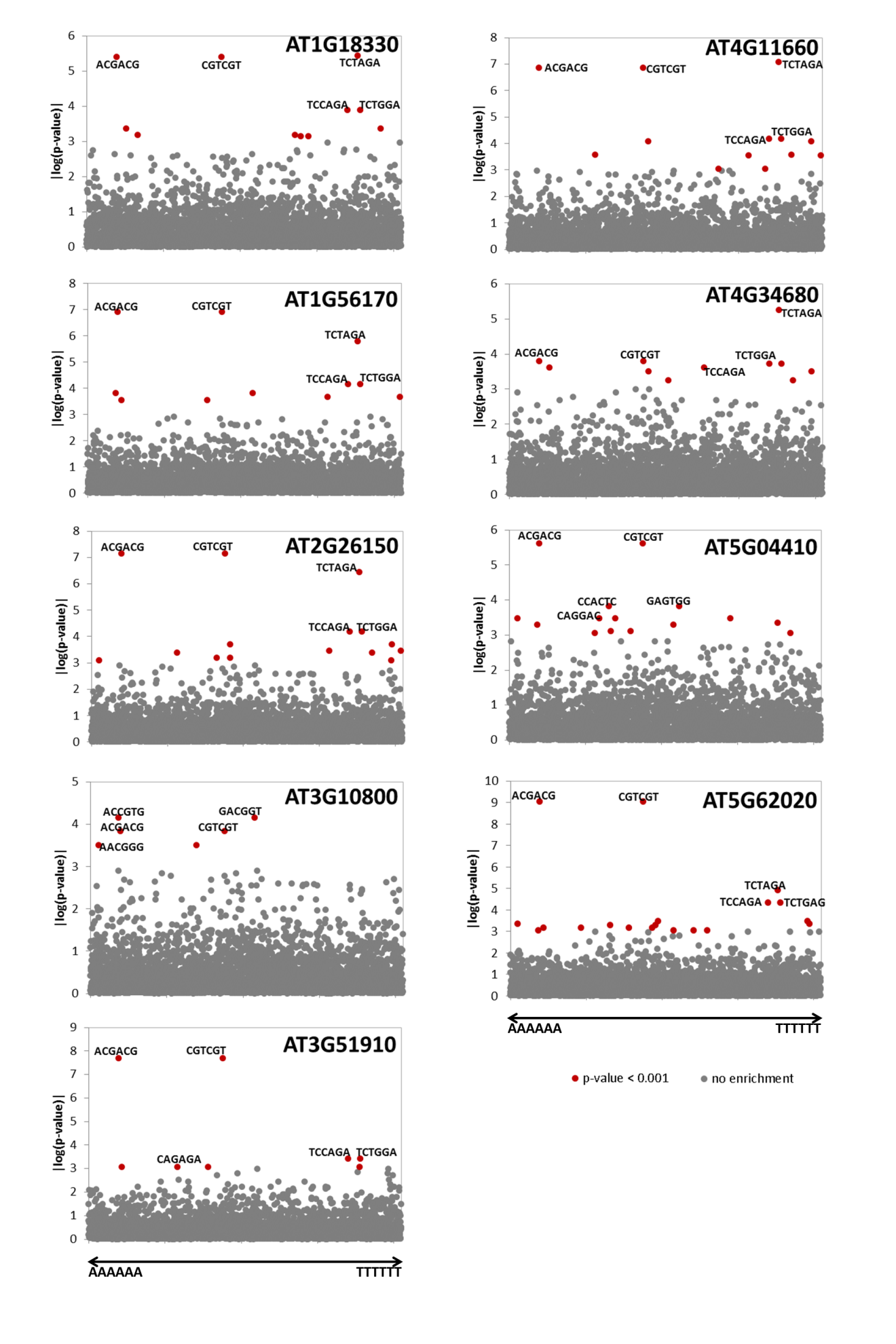


**Figure S5.** All possible 6-mer in the promoters of the co-expressed group for CsTFs under heat stress.

**
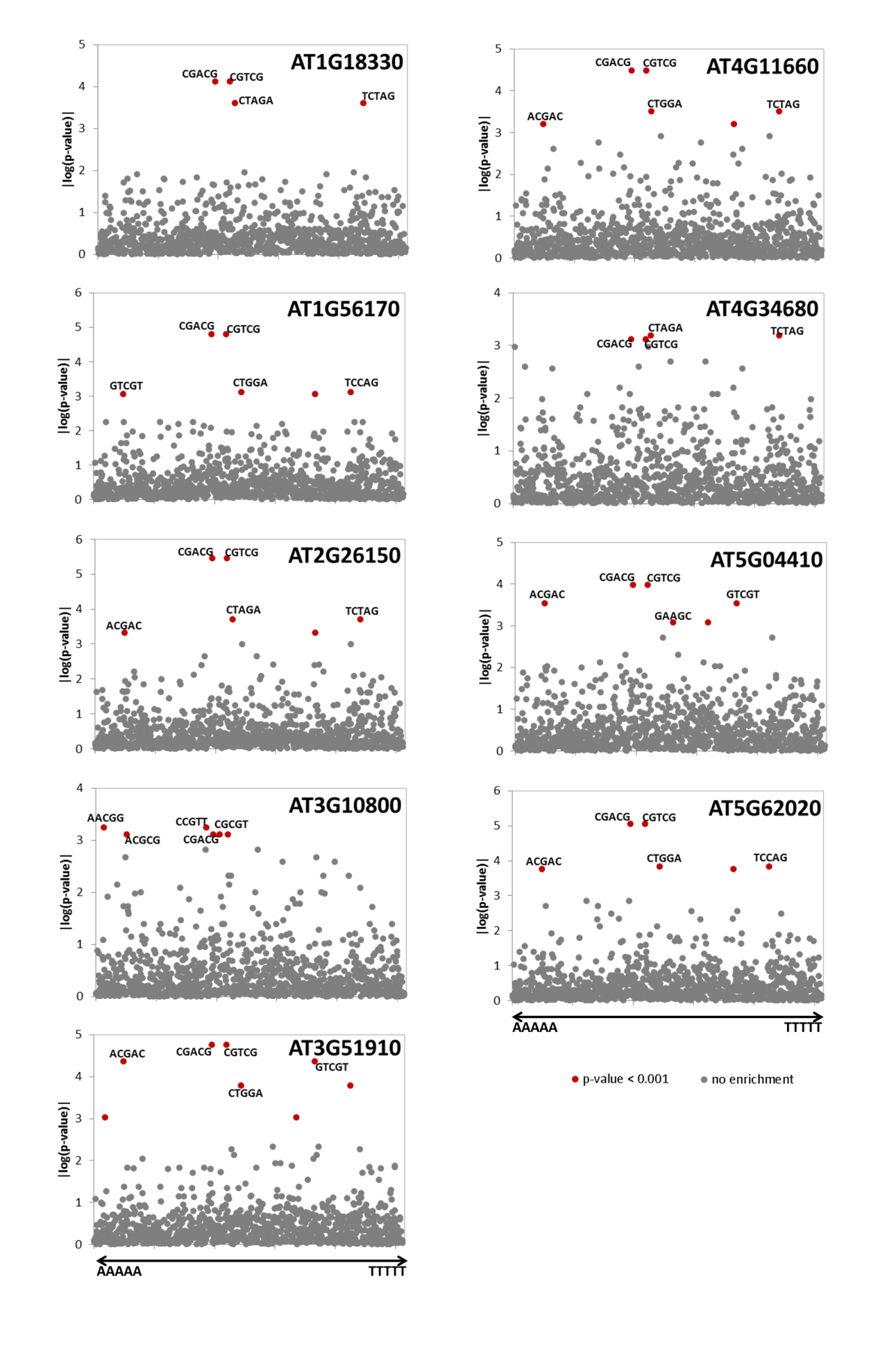
**

**Figure S6.** All possible 5-mer in the promoters of the co-expressed group for CsTFs under heat stress.


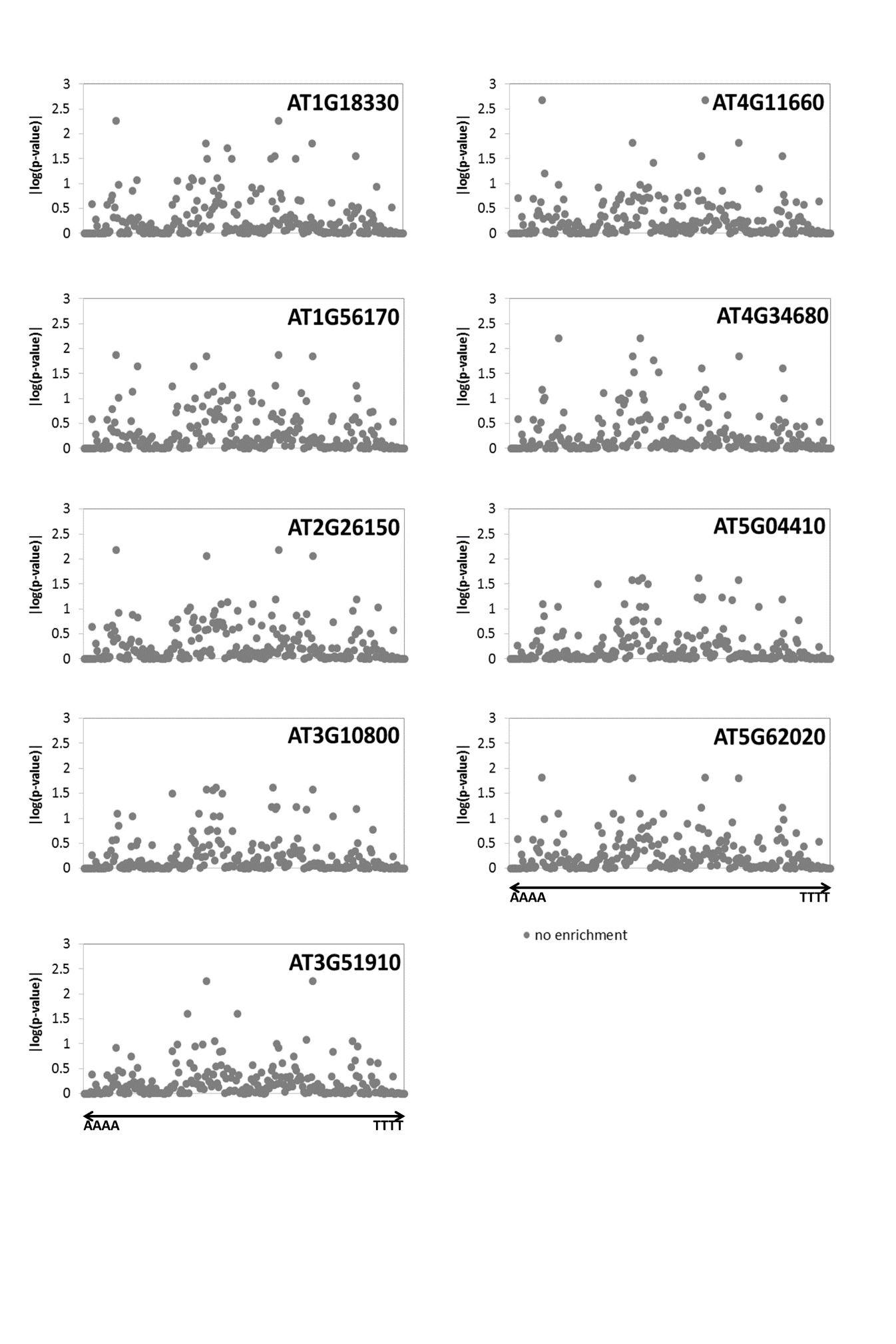


**Figure S7.** All possible 4-mer in in the promoters of the co-expressed group for CsTFs under heat stress.


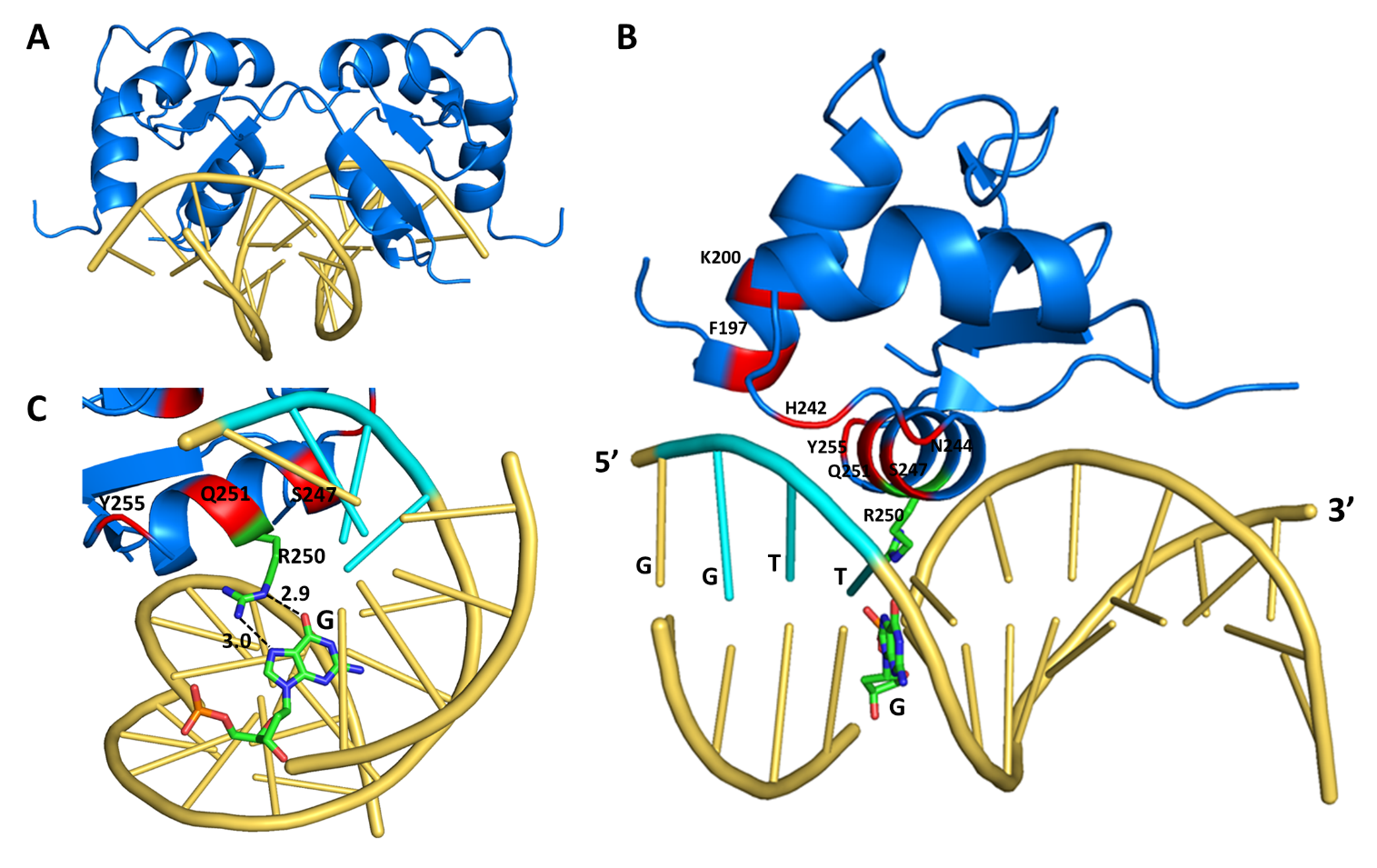


**Figure S8.** The structure of HSF-DNA complex. (A) A crystal structure shows the interface between HSF’s DNA-binding domain (DBD) (blue) and DNA (yellow) of *Kluyveromyces lactis*. DBDs of HSF bind to the DNA as a dimer by one helix of each DBD directly contacting to the major groove of DNA. The sequence of inverted repeat HSE, nGAAn, is 5’-GGTTCTAGAACC-3’. (B) The binding between DBD and HSE was stabilized by water-mediated hydrogen bonds among seven residues (red) and backbone of 5’-TTG-3’ (cyan). (C) Two hydrogen bonds between R250 and G on reverse sequence. The structure is download form PDB database (PDB ID: 3HTS ).


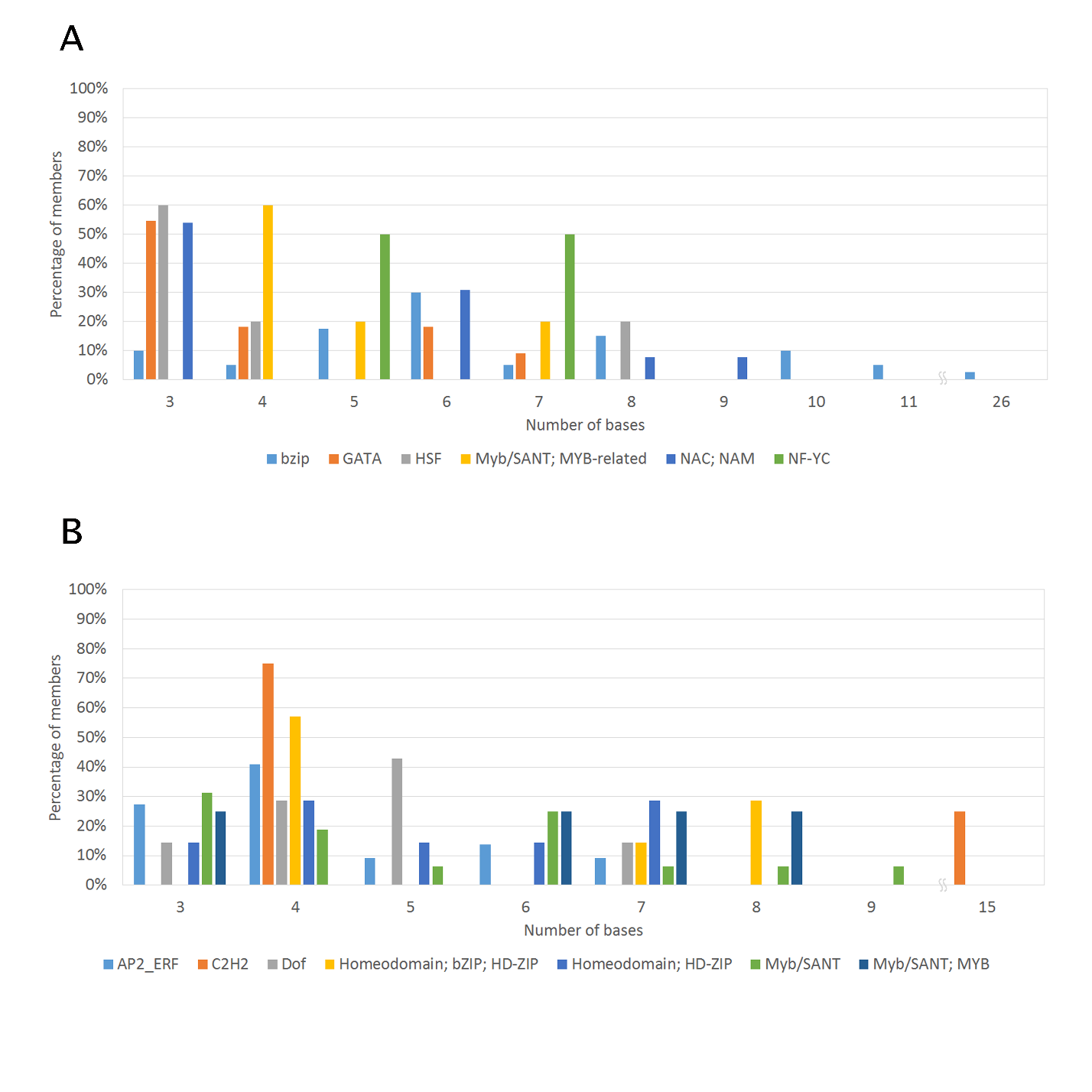


**Figure S9.** Statistics of core motifs in confirmed DNA binding matrixes for TF families. Core motifs were the continuous nucleotides with high frequency (> 70%) in position specific scoring matrices. Six families for CsTF under heat stress were shown in (A). Except for bZIP, NAC; NAM, Myb/SANT; MYB-related, NF-YC, and HSF, which have been shown in (A), seven families for ABA-CsTF were shown in (B).


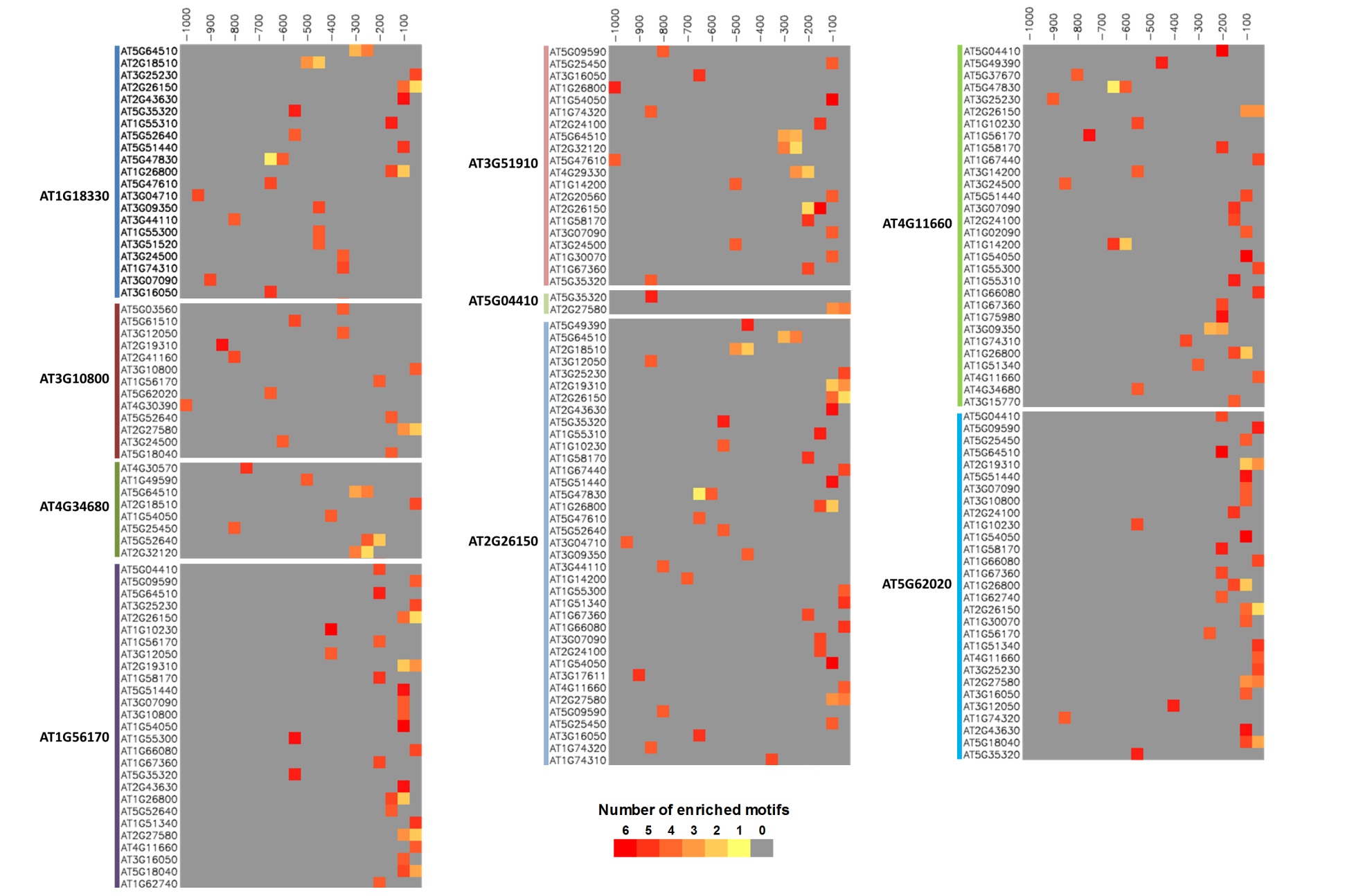


**Figure S10.** Locations for overlapping motifs in promoter regions. The right side of a row represents the transcriptional start sites, and -100 stands for upstream 100bp. The number of overlapping motifs was calculated for every 50 bases.


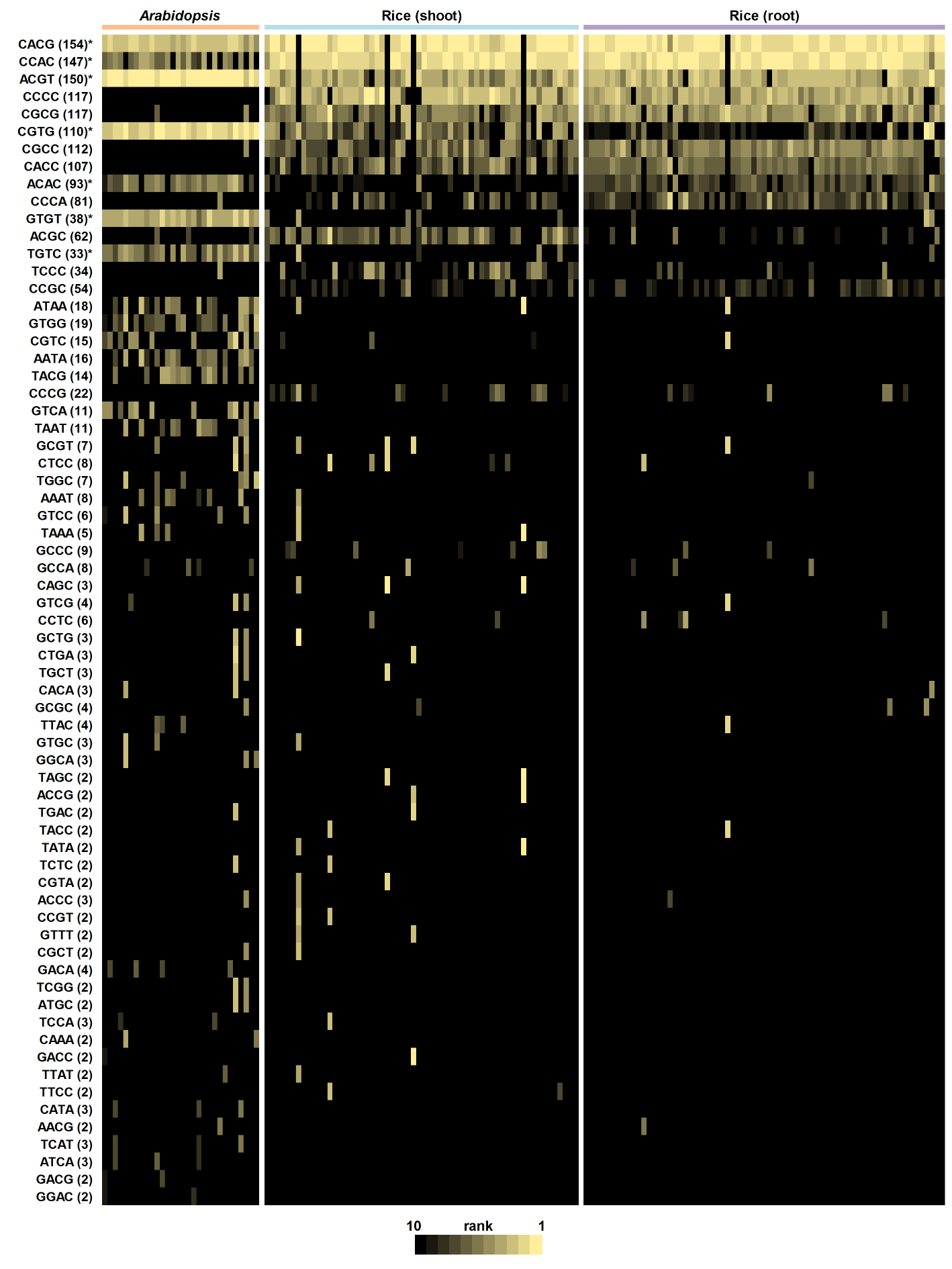


**Figure S11.** Heatmap of top 10 overrepresented 4-mer sequences for *Arabidopsis* and rice ABA-CsTFs. The number of ABA-CsTFs for each motif is in parentheses. The motifs related to ABRE are marked in starts.
